# From Prompts to Practice: Evaluating ChatGPT, Gemini, and Grok Against Plastic Surgeons in Local Flap Decision-Making

**DOI:** 10.3390/diagnostics15202646

**Published:** 2025-10-20

**Authors:** Gianluca Marcaccini, Luca Corradini, Omar Shadid, Ishith Seth, Warren M. Rozen, Luca Grimaldi, Roberto Cuomo

**Affiliations:** 1 Plastic Surgery Unit, Department of Medicine, Surgery and Neuroscience, University of Siena, 53100 Siena, Italyluca.grimaldi@unisi.it (L.G.);; 2 Faculty of Medicine and Surgery, Peninsula Clinical School, Monash University, Melbourne, VIC 3199, Australia; 3 Department of Plastic and Reconstructive Surgery, Frankston Hospital, Peninsula Health, Frankston, VIC 3199, Australia

**Keywords:** plastic surgery, local flaps, reconstructive planning, clinical images, generative AI, large language models, decision support, surgical education

## Abstract

**Background**: Local flaps are a cornerstone of reconstructive plastic surgery for oncological skin defects, ensuring functional recovery and aesthetic integration. Their selection, however, varies with surgeon experience. Generative artificial intelligence has emerged as a potential decision-support tool, although its clinical role remains uncertain. **Methods**: We evaluated three generative AI platforms (ChatGPT-5 by OpenAI, Grok by xAI, and Gemini by Google DeepMind) in their free-access versions available in September 2025. Ten preoperative photographs of suspected cutaneous neoplastic lesions from diverse facial and limb sites were submitted to each platform in a two-step task: concise description of site, size, and tissue involvement, followed by the single most suitable local flap for reconstruction. Outputs were compared with the unanimous consensus of experienced plastic surgeons. **Results**: Performance differed across models. ChatGPT-5 consistently described lesion size accurately and achieved complete concordance with surgeons in flap selection. Grok showed intermediate performance, tending to recognise tissue planes better than lesion size and proposing flaps that were often acceptable but not always the preferred choice. Gemini estimated size well, yet was inconsistent for anatomical site, tissue involvement, and flap recommendation. When partially correct answers were considered acceptable, differences narrowed but the overall ranking remained unchanged. **Conclusion**: Generative AI can support reconstructive reasoning from clinical images with variable reliability. In this series, ChatGPT-5 was the most dependable for local flap planning, suggesting a potential role in education and preliminary decision-making. Larger studies using standardised image acquisition and explicit uncertainty reporting are needed to confirm clinical applicability and safety.

## 1. Introduction

Plastic and reconstructive surgery regularly manages skin defects caused by oncological disease, trauma, or other conditions. In this field, local flaps are among the most commonly used techniques. They enable the surgeon to move adjacent tissue while maintaining reliable blood supply and providing stable coverage with colour and texture similar to the surrounding skin [1].

Choosing the most suitable flap is not always straightforward. The decision depends on various factors, including the anatomical location, size and depth of the defect, the condition of the surrounding tissues, the patient’s overall health, and the surgeon’s experience level. For the same defect, different surgeons may suggest different options, each technically correct but with aesthetic and functional implications that can differ significantly [2,3]. The literature has attempted to mitigate this variability by developing algorithms and decision-making charts, particularly for nasal, labial, cheek, and eyelid reconstruction. However, none of these systems has achieved universal standardisation, and clinical judgment remains crucial [3,4].

The training of young surgeons reflects this complexity. Learning local flap techniques still relies on mentorship in the operating theatre and direct exposure to clinical cases. This approach is practical but inconsistent and heavily influenced by the availability of cases. In some settings, the range of clinical defects may be too limited to offer comprehensive and uniform training. As a result, there is growing interest in supplementary tools that can simulate real clinical scenarios and deliver immediate feedback. At the same time, artificial intelligence has seen unprecedented growth. Large language models (LLMs) have shown the ability to understand natural language and generate complex, contextually suitable responses [4,5]. Their performance also depends greatly on how the prompt is formulated, since even small changes in the wording can lead to different interpretations and outcomes. At the same time, these models may occasionally produce information that sounds plausible but is not accurate, which underlines the importance of careful supervision and critical evaluation when using them in a medical setting [3,5]. In plastic surgery, several recent studies have explored these abilities across diverse areas, including the management of Dupuytren’s disease, microsurgical planning, breast reconstruction, burns, and hand fractures [5,6].

These studies demonstrate that AI can provide accurate responses in relatively well-defined and simple scenarios. However, its reliability often decreases in complex situations, emergencies, or when information is incomplete [6,7]. In such cases, the clinical reasoning of the surgeon, based on tacit knowledge and intraoperative sensitivity, remains essential. Therefore, artificial intelligence should not be viewed as a replacement for clinical judgment but rather as a tool that offers educational support and encourages critical reflection. One area that has received limited attention is AI’s ability to suggest reconstructive solutions for actual skin defects documented with clinical images [3,4]. This type of assessment is especially relevant because local flaps are based on geometric principles and technical rules that are well established and commonly found in surgical textbooks. The ability to submit authentic clinical images to AI systems, asking them first to describe the defect and then to recommend the most suitable flap, allows for testing not only their theoretical understanding but also their visual analysis skills and ability to translate observations into concrete surgical decisions.

Another challenge in evaluating artificial intelligence in surgery lies in the gap between experimental validation and real-world clinical practice. Most studies assessing AI performance in skin cancer or lesion recognition rely on curated dermoscopic datasets, collected under highly standardised conditions, which often inflate accuracy levels [8,9]. By contrast, plastic surgeons frequently work with photographs taken in uncontrolled environments, with variable lighting, patient positioning, and lesion presentation. These conditions reflect the complexity of daily clinical work but also represent a far more demanding test for generative AI platforms. Beyond diagnostic accuracy, reconstructive planning introduces an additional layer of difficulty, requiring the translation of image analysis into procedural choices that integrate geometry, anatomy, and surgical principles [3,4]. For this reason, evaluating AI in local flap decision-making is not only a technological exercise but also a clinically meaningful benchmark of whether these systems can truly engage with surgical reasoning.

The aims of this study are threefold. First, this study aims to assess the accuracy of AI-generated descriptions of actual clinical defects. Second, it aims to compare the reconstructive solutions suggested by ChatGPT, Grok, and Gemini with those traditionally accepted by experienced plastic surgeons. Third, it aims to evaluate the educational potential of these applications, which could serve as valuable training resources for residents and young surgeons, particularly in areas with limited access to diverse case experiences. In summary, this study goes beyond testing the theoretical consistency of AI systems. Instead, it examines their ability to engage with real clinical cases, transforming an image into a reconstructive reasoning process. This investigation was conceived as a proof-of-concept study, aimed at exploring whether current generative AI systems can translate image-based analysis into reconstructive reasoning rather than validating their clinical performance. The goal is to identify the strengths and limitations of ChatGPT, Grok, and Gemini in local flap planning and to consider their potential as future tools for both surgical education and clinical support.

## 2. Materials and Methods

The study was conducted in accordance with the principles outlined in the Declaration of Helsinki and current ethical guidelines for observational clinical research. All patients provided written informed consent for the publication of their images for scientific and educational purposes.

Clinical cases were chosen from the activity of the Plastic and Reconstructive Surgery Unit at the University Hospital Le Scotte, Siena, Italy. Ten representative cases of suspected cutaneous neoplastic lesions were included, selected to cover a range of anatomical sites frequently encountered in clinical practice, including scalp, lower lip, eyelid, malar region, nasal dorsum, frontal region, and middle third of the leg. This diversity was intended to expose the AI models to different reconstructive challenges, from highly visible cosmetic areas such as the face to more functionally oriented sites such as the leg. All cases were documented with preoperative clinical images. These photographs were deliberately not taken under standardised photographic protocols but in natural conditions, to mimic the immediacy and spontaneity of a typical clinical interaction between a surgeon and artificial intelligence.

Three generative AI platforms, tested in their publicly available versions as of 13 September 2025, were assessed: ChatGPT-5 (OpenAI, San Francisco, CA, USA), Grok (xAI, San Francisco, CA, USA), and Gemini (Google DeepMind, London, UK).These systems were selected for their broad accessibility, ease of use across devices, and the ability to interact in natural language without requiring technical training. This choice reflects the reality of how such platforms would likely be used by clinicians or trainees in everyday practice, outside of specialised or paid environments.

Each clinical case was submitted individually to the three platforms following a standardised two-step process. In the first step, the AI was asked to examine the preoperative image and provide a concise description of the lesion, including anatomical site, approximate size, and tissue involvement. A uniform prompt was applied across all platforms to minimise bias and ensure comparability of outputs. In the second step, the same image was presented again with the request to specify which local flap should be used for reconstruction, with the instruction to provide only one flap name. This sequential approach was designed to separate descriptive ability from decision-making, reflecting the logical progression of clinical reasoning.

The responses generated by the platforms were collected and compared with the decisions of a panel of experienced plastic surgeons from the same unit. The panel included both senior consultants and faculty with extensive reconstructive experience, and each case was discussed until a unanimous consensus was reached. This consensus was regarded as the gold standard, as it combined individual expertise with group deliberation to reduce subjectivity.

The data were then organised into two comparative tables. Table 1 presents the clinical descriptions provided by the artificial intelligence platforms alongside those of the surgeons’ panel. In contrast, Table 2 details the reconstructive proposals suggested by the platforms and their comparison with the consensus decision. To allow for quantitative analysis, the textual information was subsequently converted into numerical variables using a predefined coding system, enabling descriptive statistics and concordance assessment.

### Statistical Analysis

The data obtained from the AI platforms’ responses were transformed into numerical variables according to predefined coding rules. For the clinical description of the lesions (Table 2), each case was evaluated on three parameters—site, size, and tissue involvement—and scored as 0 = incorrect, 1 = partially correct, and 2 = correct. For size, a tolerance of ±0.5 cm from the longest diameter was accepted for full credit. The results were summarised as absolute frequencies and percentages of correct responses for each parameter, and 95% confidence intervals were calculated using the Wilson binomial method (Table 3 and Table 4).

For flap selection (Table 5 and Table 6), AI responses were coded similarly: 0 = inappropriate flap, 1 = partially correct or clinically acceptable but not coincident with the gold standard, and 2 = correct flap identical to the surgeons’ consensus. Absolute and percentage frequencies of entirely correct (score = 2) and at least acceptable (score ≥ 1) responses were reported, with corresponding 95% confidence intervals. In addition, the mean score (range 0–2) was calculated for each model as an overall performance index.

No comparative statistical tests between models were performed due to the limited sample size (n = 10). The analysis was therefore conducted with a descriptive and exploratory intent, aimed at assessing the relative accuracy and potential educational value of the artificial intelligence systems under investigation.

## 3. Results

The analysis of descriptive performance (Table 1) revealed striking heterogeneity in the way the three AI platforms handled real clinical images. ChatGPT emerged as the most reliable model when it came to estimating lesion size, correctly identifying the dimension in 90% of cases (95% CI 59.6–98.2). This ability to approximate size with high consistency mirrors one of the most fundamental steps in clinical evaluation. However, its performance dropped considerably in other domains: anatomical site was correctly identified in only 40% of cases (95% CI 16.8–68.7), and tissue involvement was accurately described in just 10% (95% CI 1.8–40.4). These figures suggest that while ChatGPT can process geometric aspects with reasonable precision, it struggles when confronted with subtler anatomical or histological nuances. The quantitative accuracy scores for each parameter are summarised in Table 3 and Table 4.

Gemini displayed a broadly similar pattern but with slightly weaker overall performance. Like ChatGPT, it was strong in estimating lesion size (90%, 95% CI 59.6–98.2), yet accuracy fell when dealing with localisation (30%, 95% CI 10.8–60.3) and tissue involvement (20%, 95% CI 5.7–51.0). This tendency highlights a recurring limitation of the model: the ability to grasp measurable parameters, such as dimensions, contrasts with difficulties in interpreting relational or contextual features that are often critical in surgical planning.

Grok, on the other hand, showed a different distribution of strengths and weaknesses. Its performance in identifying site (30%, 95% CI 10.8–60.3) and size (50%, 95% CI 23.7–76.3) was modest compared with the other two models. Interestingly, however, Grok outperformed both ChatGPT and Gemini in recognising tissue depth, correctly describing planes in 60% of cases (95% CI 31.3–83.2). This suggests that Grok, although less consistent overall, may possess a relative advantage in aspects of visual interpretation that involve stratification and deeper layers.

When the threshold for evaluation was broadened to include not only fully correct answers but also partially acceptable ones (score ≥ 1), all platforms improved their outcomes. Under these more lenient conditions, ChatGPT achieved 80% accuracy for site, 100% for size, and 90% for tissues. Gemini performed similarly well, with 90% for both site and size and 80% for tissues. Grok’s performance was less balanced, 60% for site, 50% for size, and 70% for tissues, but still reflected its comparative strength in evaluating tissue planes. These findings indicate that while ChatGPT and Gemini are more dependable in straightforward tasks such as size estimation, Grok’s advantage lies in tackling one of the most challenging dimensions of image analysis: the depth of involvement.

Moving to flap selection (Table 5), the contrasts among the platforms became even more pronounced. ChatGPT achieved complete concordance with the surgeons’ consensus, identifying the correct flap in every single case (100%, 95% CI 72.2–100.0). This level of accuracy is notable because flap selection integrates multiple layers of reasoning, from the anatomical and geometric to the functional and aesthetic, making it a demanding test of clinical understanding. Grok reached 70% accuracy (95% CI 39.7–89.2), a respectable but clearly lower performance, while Gemini lagged behind, providing the correct choice in only 20% of cases (95% CI 5.7–51.0). Detailed scoring of flap concordance is reported in Table 6 and Table 7.

Once again, considering partially acceptable answers improved the overall picture. ChatGPT maintained full agreement at 100%, confirming the robustness of its outputs. Grok improved to 90% (95% CI 59.6–98.2), reflecting that while not always exact, its proposals were often clinically reasonable. Gemini rose to 40% (95% CI 16.8–68.7), yet its low concordance under strict criteria underscores its current limitations in reconstructive decision-making.

Average performance scores (0–2 scale) further highlighted this hierarchy: 2.00 for ChatGPT, 1.60 for Grok, and 0.60 for Gemini. The gap between ChatGPT and the other two platforms reflects not only quantitative differences but also a qualitative divide in their ability to integrate descriptive accuracy into practical reconstructive reasoning.

Taken together, these results confirm the considerable variability that exists among current AI platforms in their capacity to support surgical tasks. ChatGPT consistently stood out as the most reliable, capable of matching expert surgeons across both descriptive and decision-making processes. Grok, although less precise, demonstrated valuable competence in tissue recognition, suggesting potential for targeted refinement in this area. Gemini, while showing promise in size estimation, struggled to extend this strength into other critical aspects of analysis. These findings illustrate both the potential and the current limitations of generative AI in reconstructive surgery: a tool that can already provide valuable insights but still requires refinement and validation before widespread clinical integration.

## 4. Discussion

The analysis carried out in this study revealed notable variability among artificial intelligence platforms in both describing cutaneous lesions and proposing reconstructive solutions with local flaps. ChatGPT proved to be the most consistent model, achieving complete agreement with the surgeons’ consensus in flap selection and demonstrating the highest accuracy in estimating lesion size, while maintaining acceptable reliability in site and tissue identification. Grok provided intermediate results, with relatively stronger performance in tissue involvement but lower accuracy in flap choice and size estimation. Gemini, although capable of reaching high accuracy in estimating lesion dimensions, showed significantly lower reliability for both anatomical site and tissue assessment, as well as limited agreement in flap selection. This pattern of relative superiority of ChatGPT over Gemini is not limited to reconstructive plastic surgery, but also resonates with evidence emerging from other surgical fields. Carlà et al. [10] demonstrated that ChatGPT-4 achieved substantially higher concordance than Gemini with vitreoretinal surgeons in planning retinal detachment procedures, not only in terms of agreement but also in quality-of-reasoning scores. Similarly, Kim and Kim [11] showed that ChatGPT markedly outperformed Gemini in diagnosing radiolucent jaw lesions, particularly when multimodal data were provided, reaching accuracy levels far above those of Gemini. Together, these studies reinforce the impression that ChatGPT currently offers more clinically aligned and reproducible outputs than competing platforms across different surgical domains. The observed heterogeneity among models has clear practical implications. ChatGPT excelled in reasoning and flap planning, while Grok performed better in tissue recognition. This fragmentation underscores the impracticality of using multiple AI systems for different steps of one surgical decision and the need for a unified, multimodal model capable of consistent support throughout reconstructive planning. Because these systems rely entirely on language, their accuracy also depends on the precise use of medical terminology. We noted occasional misuse or oversimplification of technical terms, which can affect the interpretation of complex reconstructive concepts.

These findings are especially important when compared with existing literature. Several studies have documented artificial intelligence’s potential in recognising skin tumours from clinical or dermoscopic images, reporting very high but varied accuracy levels. Nawaz et al. [9], for example, showed that optimised CNNs trained on large, balanced datasets can reach an accuracy of up to 96 per cent, surpassing established pre-trained models. Yuan et al. [12] demonstrated that semi-supervised methods, even with only a few labelled images, can sustain acceptable performance (77%), making their application more feasible in clinical settings where labelled data are limited.

However, our findings partly differ from these experiences, showing lower accuracy levels, especially for anatomical sites and tissue involvement. The methodological features of our study can explain this difference. The images used were not from standardised dermatological datasets, but from preoperative clinical photographs taken in real-world conditions, with variable quality and no uniform acquisition guidelines. Far from being a limitation, this choice reflects the daily reality faced by plastic surgeons, who often have to interpret non-standardised images. This approach probably reduced the platforms’ accuracy compared to experimental studies based on controlled datasets [3,8], but it offered a more authentic view of clinical practice. Another aspect that emerges from the comparison with the literature is the issue of transparency and interpretability. Hauser et al. [3], in a systematic review, emphasised that deep learning models, despite their high performance, remain essentially “black boxes,” with decision-making processes that are not explainable and limit clinical trust. This element is particularly relevant in light of our results. ChatGPT’s ability to consistently provide the correct answer cannot be interpreted as synonymous with absolute reliability, since it is not possible to trace the logical reasoning behind the answer. Lack of interpretability, therefore, remains a crucial obstacle for safe clinical integration. Moreover, the absence of confidence scores or uncertainty estimates makes these systems ethically and clinically unsuitable for unsupervised decision-making, as previously highlighted in Bayesian frameworks addressing overconfident AI outputs [7]. Comparable concerns have been raised in oncology: Hernández-Flores et al. [13] reported only moderate agreement between ChatGPT and multidisciplinary tumor boards for surgical and radiotherapy decisions, while Gemini showed merely fair concordance, highlighting the persistent risk of misalignment with expert opinion. Likewise, Lorenzi et al. [14] observed that ChatGPT-4 produced more guideline-concordant treatment plans than Gemini in head and neck oncology, yet disagreement rates remained significant. These studies collectively confirm that, despite relative superiority, ChatGPT must still be regarded as a tool requiring careful clinical oversight.

Other studies have emphasised the importance of quantifying uncertainty. Abdar et al. [7], for example, introduced Bayesian frameworks to prevent overconfident misclassifications, demonstrating that incorporating uncertainty estimation reduces error risks. This is also indirectly evident in our study. The platforms consistently delivered definitive responses without indicating a level of confidence, which can be a significant limitation for clinical applications. Another element of novelty lies in the ability to propose reconstructive solutions. While most of the available literature focuses on the diagnostic recognition of cutaneous lesions [15,16,17], very few studies have explored the role of AI in suggesting reconstructive strategies. In this sense, our work presents an original approach, testing the ability of the platforms to integrate lesion description with the selection of the most appropriate local flap. It is noteworthy that ChatGPT achieved 100% concordance with the surgical gold standard, a result that far exceeds the current evidence [18,19]. Comparable trends have recently been described in oncology, where ChatGPT has been shown to approach multidisciplinary decision-making standards: Li et al. [18] documented significantly higher accuracy and completeness of ChatGPT-4o compared with Gemini Advanced in the management of advanced gastric cancer, and Choo et al. [20] found an impressive 86.7% concordance rate between ChatGPT-derived colorectal cancer management plans and institutional tumor boards. These findings suggest that the superiority of ChatGPT in our series is not an isolated observation, but part of a broader and cross-specialty pattern. Similarly, Karampinis et al. recently demonstrated that large language models can also serve as effective educational tools in dermatology, supporting less experienced physicians in diagnostic reasoning and case-based learning [21].

For comparison, De La Hoz et al. (2024) [2] documented the use of CNNs for intraoperative perforator vessel identification in free flap reconstruction, reporting high accuracy levels but limited to the microsurgical context. Similarly, Thamm et al. [1] highlighted the role of AI in supporting microsurgery and robotics, enhancing precision and preoperative analysis, but with supportive rather than substitutive functions. In this regard, our findings extend the field by suggesting that generative platforms can already provide consistent responses in planning local flaps, though with necessary caution. The international literature also emphasises how dataset quality and heterogeneity strongly influence AI performance. Studies such as those by Huynh et al. [6] and Abd Elaziz et al. [17] have demonstrated that advanced class balancing, feature engineering, and architectural optimisation can considerably improve accuracy. Our decision to use non-standardised clinical images and the basic versions of the platforms (not trained explicitly for medical purposes) thus presents a “realistic” test that necessarily reveals the limitations of the models but offers a clearer picture of what a surgeon might face when directly engaging with AI.

Finally, the limitations of our study must be recognised. The small number of cases (n = 10) limits broad generalisations, and the lack of prospective follow-up prevents assessment of the actual clinical impact of the reconstructive options suggested by AI. Given the limited sample size, the apparent 100% concordance observed for ChatGPT should be interpreted as indicative rather than conclusive. A larger and more diverse dataset could yield different performance rankings, and the present findings should therefore be viewed as a proof of concept rather than evidence of established clinical reliability. Additionally, the absence of confidence quantification by the platforms and the generic nature of some outputs (particularly from Grok and Gemini) highlight the need for further development before they can be used safely in clinical settings. Nonetheless, these limitations present opportunities for future research, which should include multicentre studies, larger case series, and standardised protocols for interaction with AI.

## 5. Conclusions

This study demonstrates that current AI platforms show heterogeneous accuracy when applied to the description of cutaneous lesions and the selection of local flaps. Among the systems evaluated, ChatGPT emerged as the most consistent, achieving complete concordance with expert surgeons in reconstructive planning and showing superior performance in lesion size assessment, although less reliable in localisation and tissue depth. Grok displayed partial reliability, with its relative strength in recognising tissue planes but a weaker performance in flap selection, while Gemini, despite accurate estimation of lesion size, showed limited accuracy across most other domains. These findings, although preliminary and based on a small series of clinical cases, suggest that generative AI has the potential to serve as an adjunctive tool in plastic and reconstructive surgery, particularly in the context of surgical education and decision support. The possibility of exposing AI platforms to authentic, non-standardised clinical images and observing their reasoning process highlights their capacity to simulate aspects of surgical thought, offering structured feedback that may benefit residents and junior surgeons. At the same time, the variability observed between platforms underlines the current limitations of these technologies and the need for cautious interpretation of their outputs. Future studies should involve larger case series, standardised image acquisition, and multicentre validation to better define accuracy, reproducibility, and clinical safety. These results should be regarded as preliminary and hypothesis-generating. The perfect agreement observed in this small sample does not imply consistent reproducibility in larger or more heterogeneous clinical contexts. Although these tools cannot replace expert decision-making, their suggestions may still influence the surgeon’s reasoning process, particularly in borderline or uncertain cases. This underlines the need for critical evaluation of AI outputs and for maintaining full clinical responsibility in every decision. Ultimately, generative AI cannot substitute for surgical expertise and judgment, but it may represent a valuable complement capable of fostering critical reflection, supporting the training of young surgeons, and potentially contributing to more standardised and transparent decision-making processes in reconstructive practice.

## Figures and Tables

**Table 1:** Comparison of clinical descriptions provided by ChatGPT, Grok, and Gemini after viewing the preoperative images, using the following standardized prompt: “Look carefully at the preoperative clinical image provided. Describe only the skin lesion or skin defect, reporting the anatomical location, approximate size, and tissue involvement. Write only the description, without introductory words, comments, or explanations. Provide the answer in one concise sentence.” The surgeons’ consensus description was used as the gold standard.

Clinical Case	ChatGPT Response	Gemini Response	Grok Response	Surgeons’ Consensus Description
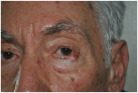	Small post-surgical skin defect of approximately 0.5–0.7 cm at the medial third of the right lower eyelid margin, involving epidermis and dermis with partial eyelid contour irregularity.	A 0.5 cm ulcerated lesion with rolled borders and surrounding erythema and telangiectasias located on the lateral aspect of the left lower eyelid margin, involving the skin and underlying tarsal plate.	The ulcerated skin lesion is located on the right upper eyelid, measuring approximately 0.5 cm in greatest dimension, and involves partial-thickness skin with surrounding erythema.	Nodular lesion measuring approximately 0.5 cm in diameter, showing superficial ulceration and telangiectasias, with poorly defined margins in the upper portion and pearly, elevated borders in the lower portion; located at the lateral canthus of the left lower eyelid; with involvement of the conjunctiva and probable extension to the dermis.
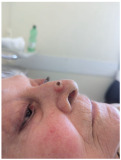	Solitary, exophytic, pigmented nodular lesion measuring approximately 6–7 mm in diameter located on the nasal tip, limited to the cutaneous and subcutaneous tissue without deeper structural involvement.	A single, dome-shaped, and hyperpigmented nodule is located on the tip of the nose, measuring approximately 0.8 cm in diameter, with full-thickness involvement of the skin.	The skin lesion is a small, dark, crusted nodule approximately 0.5 cm in diameter located on the side of the nose, involving the epidermis and possibly the upper dermis.	Exophytic nodular cutaneous lesion, measuring approximately 0.5 cm in diameter, with a crusted apical surface and well-defined, erythematous margins; located just superior to the right nasal ala; with probable invasion of the superficial dermis only.
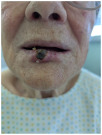	Ulcerated, exophytic, pigmented-crusted lesion measuring approximately 15–18 mm in diameter located on the midline of the lower lip, involving the vermilion border and adjacent cutaneous tissue.	A solitary, exophytic, ulcerated, and crusted lesion is present on the central lower lip, measuring approximately 1.5 cm in diameter, with full-thickness involvement of the lip vermilion and extending to the surrounding skin.	The skin lesion is a dark, crusted, ulcerative mass approximately 1 cm in diameter located on the upper lip, involving the epidermis and possibly the dermis.	Exophytic and ulcerated cutaneous lesion of oval shape, with central crust and a maximum diameter of approximately 2 cm, exhibiting ill-defined erythematous margins; located at the mid-portion of the lower lip, involving the vermilion with probable extension to subcutaneous and submucosal tissues and adjacent perilesional skin.
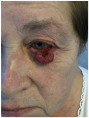	Ulcerated nodular lesion of approximately 2.5–3 cm located at the left infraorbital region, involving the lower eyelid margin and extending to the medial canthus with partial distortion of the eyelid contour.	A well-defined, multi-lobulated, flesh-colored, friable mass of approximately 2 cm in diameter, with central ulceration and surrounding telangiectasias, located on the medial aspect of the left lower eyelid, involving the eyelid margin and extending into the conjunctival tissue.	The ulcerated skin lesion is located on the left lower eyelid, measuring approximately 1 cm in greatest dimension, and involves full-thickness skin with exposed subcutaneous tissue and surrounding erythema.	Elevated nodular lesion, reddish-violaceous in color, measuring approximately 3 cm in diameter, with a crusted area and central ulceration showing a necrotic and exudative base; located in the mid-portion of the left lower eyelid, with involvement of the eyelid margin and conjunctiva.
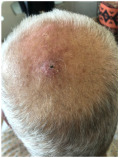	Erythematous nodular lesion with central ulceration and crusting, approximately 10–12 mm in diameter, located on the vertex scalp, involving skin and superficial subcutaneous tissue.	A nodular and ulcerated lesion is present on the scalp, measuring approximately 1.5 cm in diameter, with full-thickness skin involvement.	The skin lesion is a small, dark, crusted ulcer approximately 0.5 cm in diameter located on the top of the scalp, involving the epidermis and possibly the upper dermis.	Nodular cutaneous lesion with central ulceration, measuring approximately 1.5 cm, with irregular yet well-defined margins; located at the vertex of the scalp, with probable invasion of the upper dermis but without apparent involvement of the surrounding skin.
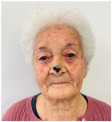	Ulcerated, exophytic, necrotic-crusted lesion measuring approximately 20–25 mm in diameter located on the nasal tip and columella, involving cutaneous and subcutaneous tissues with partial obstruction of the nostrils.	A necrotic lesion is present on the nasal dorsum and tip, measuring approximately 2.0 × 2.5 cm, with full-thickness involvement of the skin, including cartilage.	The skin lesion is a dark, crusted, ulcerative mass approximately 1.5 cm in diameter located on the tip of the nose, involving the epidermis, dermis, and possibly underlying cartilage.	Exophytic crusted cutaneous lesion with hyperkeratotic and necrotic features, rhomboid in shape, measuring approximately 2 × 2 cm, with well-defined margins and no apparent involvement of the surrounding skin; located at the tip of the nose; involvement probably limited to subcutaneous tissue.
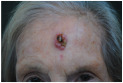	Ulcerated nodular lesion with central necrosis and peripheral induration, approximately 15–18 mm in diameter, located on the central forehead, involving cutaneous and superficial subcutaneous tissue.	A centrally ulcerated and crusted lesion with a raised erythematous border is located on the central forehead, measuring approximately 2.0 cm in diameter, with full-thickness involvement of the skin.	The skin lesion is a dark, crusted, ulcerative mass approximately 1.5 cm in diameter located on the center of the forehead, involving the epidermis and possibly the dermis.	Multinodular cutaneous lesion measuring approximately 2 cm in maximum diameter, characterized by a necrotic-crusted area on the superior portion and a hyperkeratotic component on the left side, with indistinct erythematous margins and a peripheral pearly rim; located on the central forehead; with probable full-thickness skin involvement.
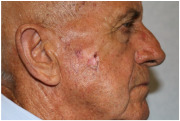	Atrophic, erythematous lesion with central ulceration and crusting, approximately 10–12 mm in diameter, located on the left malar region, involving cutaneous and superficial subcutaneous tissue.	A pinkish, irregular, and centrally ulcerated skin defect is located on the right cheek, measuring approximately 1.5 × 1.0 cm, with full-thickness involvement of the skin.	The skin lesion is a pinkish, ulcerated defect with central crust and exudate approximately 2 cm in diameter located on the cheek, involving the epidermis and dermis.	Flat cutaneous lesion measuring approximately 1 cm, with ulceration in the superior portion, a centrally atrophic and depressed area, and inferior hyperkeratosis; margins are slightly elevated, irregular, and ill-defined. Located over the right zygomatic region, with probable involvement of the deep dermis. Superoposteriorly, a smaller lesion with similar characteristics is also visible.
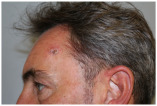	Crusted ulcerated lesion measuring approximately 8–10 mm in diameter located on the left temporal region, involving cutaneous and superficial subcutaneous tissue.	A nodular, centrally ulcerated lesion is located on the right frontal scalp, measuring approximately 1.5 cm in diameter, with full-thickness involvement of the skin.	The skin lesion is a small, crusted, erythematous ulcer approximately 1 cm in diameter located on the forehead near the hairline, involving the epidermis and possibly the upper dermis.	Flat, hyperkeratotic skin lesion measuring approximately 1.5 cm in its largest diameter, with irregular and ill-defined margins. Located on the left temporal region; with probable involvement of the dermis. Perilesional skin demonstrates the presence of telangiectasias.
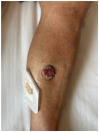	Ulcerated lesion with granulation tissue and peripheral induration, approximately 25–30 mm in diameter, located on the anterior aspect of the lower leg, involving skin and subcutaneous tissue.	A solitary, exophytic, ulcerated, and friable lesion is located on the anterior aspect of the lower leg, measuring approximately 2.0 × 2.5 cm, with full-thickness involvement of the skin.	The image shows a skin lesion on the lower leg, approximately 2 cm in diameter, appearing as a reddish, ulcerated, and crusted area, possibly involving the epidermis and dermis, with a bandage nearby.	Exophytic, ulcerated cutaneous lesion of rounded shape, measuring approximately 3 cm in diameter, with peripheral fibrinous areas and raised, irregular margins. Located on the antero-medial aspect of the left leg; with probable full-thickness skin involvement. Surrounding skin is mildly erythematous.

**Table 2:** Structured accuracy of clinical descriptions by AI model. Legend. Site, Size, and Tissues were coded against the surgeons’ consensus. Each item was scored 0 = incorrect, 1 = partially correct, 2 = correct. For Size, a tolerance of ±0.5 cm on the longest diameter was required to score 2; 1 was assigned when a numeric estimate was outside ±0.5 cm but within ±1.0 cm, or when a non-numeric category matched the correct size band; 0 otherwise. Site 2 = correct region and side; 1 = correct region but wrong subsite/side; 0 = wrong region. Tissues 2 = all involved planes, none extraneous; 1 = misses a non-critical plane or adds a minor one; 0 = misses a critical plane or adds a clearly wrong structure.

Case	ChatGPT			Gemini			Grok		
	Site	Size	Tissue	Site	Size	Tissue	Site	Size	Tissue
1	0	2	2	2	2	0	0	2	1
2	1	2	1	1	2	1	1	2	2
3	2	2	1	2	2	1	0	0	0
4	1	2	0	1	0	2	1	0	0
5	2	2	1	1	2	1	2	0	2
6	1	2	1	1	2	0	2	2	0
7	2	2	1	2	2	1	2	2	2
8	0	2	1	1	2	1	0	0	2
9	2	1	1	0	2	1	1	2	2
10	1	2	1	1	2	2	0	0	2

**Table 3:** Comparative accuracy of ChatGPT, Gemini, and Grok in describing skin defects. Score = 2.

AI	Parameter	Correct	% Correct	95%CI Low	95%CI High
ChatGPT	Site	4	40	16.8	68.7
ChatGPT	Size	9	90	59.6	98.2
ChatGPT	Tissues	1	10	1.8	40.4
Gemini	Site	3	30	10.8	60.3
Gemini	Size	9	90	59.6	98.2
Gemini	Tissues	2	20	5.7	51.0
Grok	Site	3	30	10.8	60.3
Grok	Size	5	50	23.7	76.3
Grok	Tissues	6	60	31.3	83.2

**Table 4:** Comparative accuracy of ChatGPT, Gemini, and Grok in describing skin defects. Score ≥ 1.

AI	Parameter	Correct	% Correct	95%CI Low	95%CI High
ChatGPT	Site	8	80	49.0	94.3
ChatGPT	Size	10	100	72.2	100
ChatGPT	Tissues	9	90	59.6	98.2
Gemini	Site	9	90	59.6	98.2
Gemini	Size	9	90	59.6	98.2
Gemini	Tissues	8	80	49.0	94.3
Grok	Site	6	60	31.3	83.2
Grok	Size	5	50	23.7	76.3
Grok	Tissues	7	70	39.7	89.2

**Table 5:** Comparison of local flap choices proposed by ChatGPT-5, Grok, and Gemini after viewing the preoperative images, using the following standardized prompt: “Look carefully at the preoperative clinical image provided. Indicate which local flap you would use for reconstruction. Write only the name of the flap, without explanations, comments, or alternative options. Provide only one answer.” The surgeons’ consensus flap was used as the gold standard.

Clinical Case	ChatGPT Response	Gemini Response	Grok Response	Surgeons’ Consensus Description
1	Tenzel flap	Tenzel semicircular flap	Tenzel flap	Tenzel flap
2	Bilobed flap	Dorsal nasal flap	Bilobed flap	Bilobed flap
3	Abbe flap	Abbe flap	Abbe flap	Abbe flap
4	Mustardé flap	Cutler–Beard bridge flap	Mustardé flap	Mustardé flap
5	Rotation flap	V-Y advancement flap	Rotation flap	Pinwheel rotation flap
6	Forehead flap	Medial cheek V-Y advancement flap	Bilobed flap	Forehead flap
7	A-to-T advancement flap	Glabellar flap	A-T flap	A-to-T flap
8	Limberg flap	Mustardé flap	Rhomboid flap	Limberg flap
9	O-to-H flap	Rhomboid flap	Note flap	O-to-H flap
10	Keystone flap	Fasciocutaneous propeller flap	Advancement flap	Keystone flap

**Table 6:** Local flap choices proposed by the AI models compared with the surgeons’ consensus. Each case was scored numerically: 0 = incorrect flap, 1 = partially correct flap, 2 = correct flap.

Case	ChatGPT	Gemini	Grok
1	2	2	2
2	2	1	2
3	2	2	2
4	2	0	2
5	2	0	2
6	2	0	0
7	2	0	2
8	2	0	2
9	2	0	1
10	2	1	1

**Table 7:** Accuracy of flap selection by AI models compared with surgeons’ standards.

Model	n Cases	n Correct (Score = 2)	% Correct (Score = 2)	95% CI (Score = 2) Low	95% CI (score = 2) High	n ≥ 1	% ≥1	95% CI (≥1) Low	95% CI (≥1) High	Mean Score (0–2)
ChatGPT	10	10	100	72.2	100	10	100	72.2	100	2.0
Gemini	10	2	20	5.7	51	4	40	16.8	68.7	0.6
Grok	10	7	70	39.7	89	9	90	59.6	98.2	1.6

## Data Availability

All data supporting the findings of this study are available within the article itself. No additional datasets were generated or analyzed.

## Abbreviations

The following abbreviations are used in this manuscript:

AI	Artificial Intelligence
LLM	Large Language Model
TLA	Three-letter acronym
